# Supplementary material for: Pectolinarin Inhibits the Bacterial Biofilm Formation and Thereby Reduces Bacterial Pathogenicity
Source: Antibiotics (Basel). 2022 Apr 29;11(5):598. doi: 10.3390/antibiotics11050598 (PMC9137516; doi:10.3390/antibiotics11050598)
Supplement: Supplementary file 1 [file antibiotics-11-00598-s001.zip › antibiotics-1683620-supplementary.pdf]

(a)

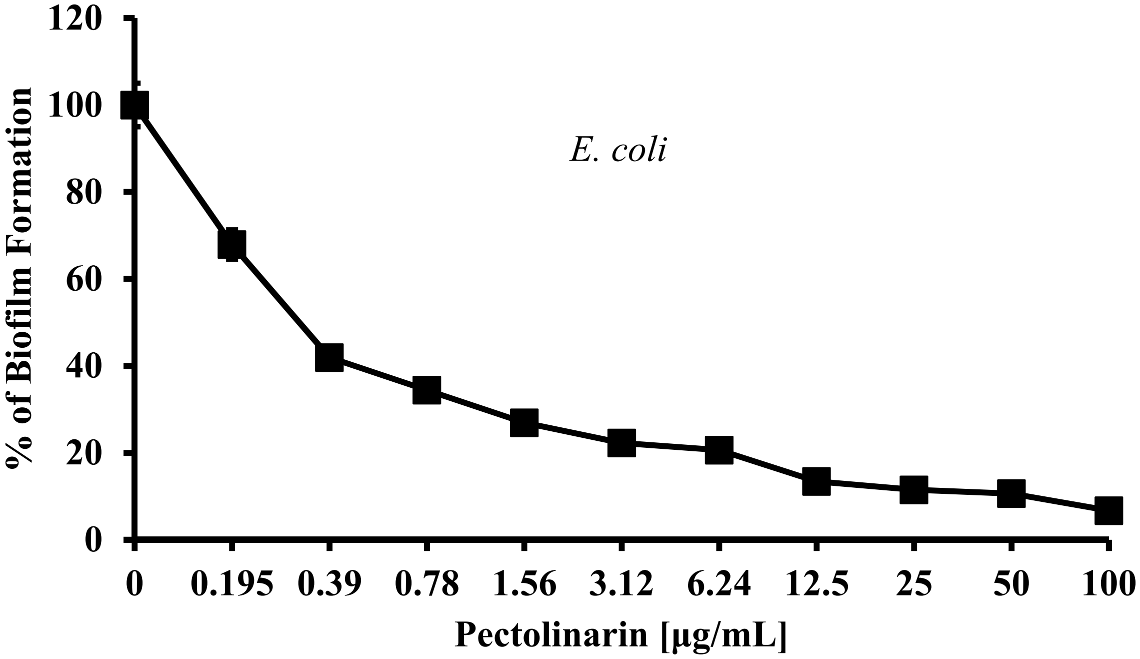
120
100
80
60
40
20
0
% of Biofilm Formation
E. coli
0
0.195
0.39
0.78
1.56
3.12
6.24
12.5
25
50
100
Pectolinarin [µg/mL]

(b)

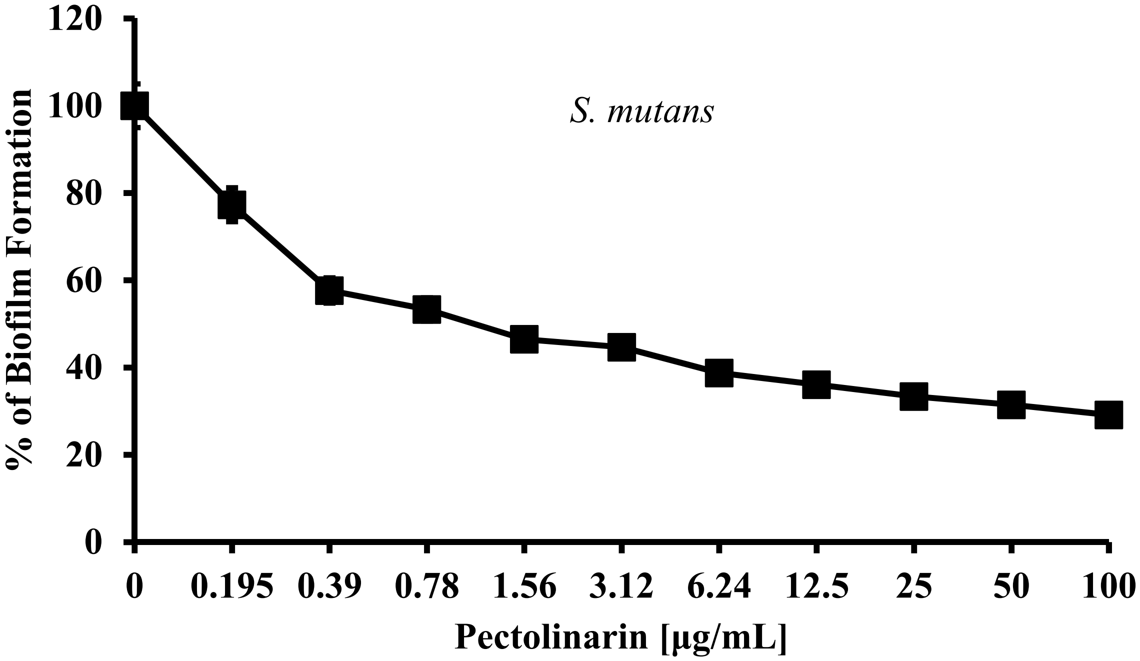
120
100
80
60
40
20
0
% of Biofilm Formation
S. mutans
0
0.195
0.39
0.78
1.56
3.12
6.24
12.5
25
50
100
Pectolinarin [µg/mL]

(c)

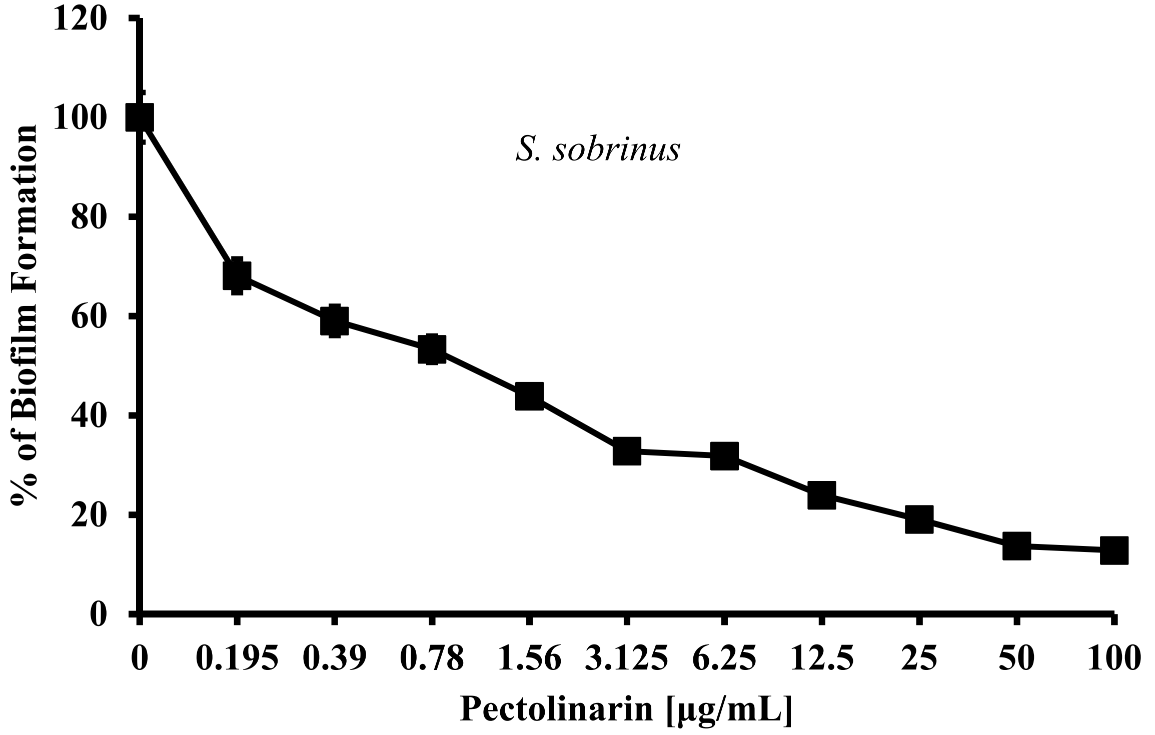

S. sobrinus
% of Biofilm Formation
120
100
80
60
40
20
0
0 0.195 0.39 0.78 1.56 3.125 6.25 12.5 25 50 100
Pectolinarin [µg/mL]


(d)

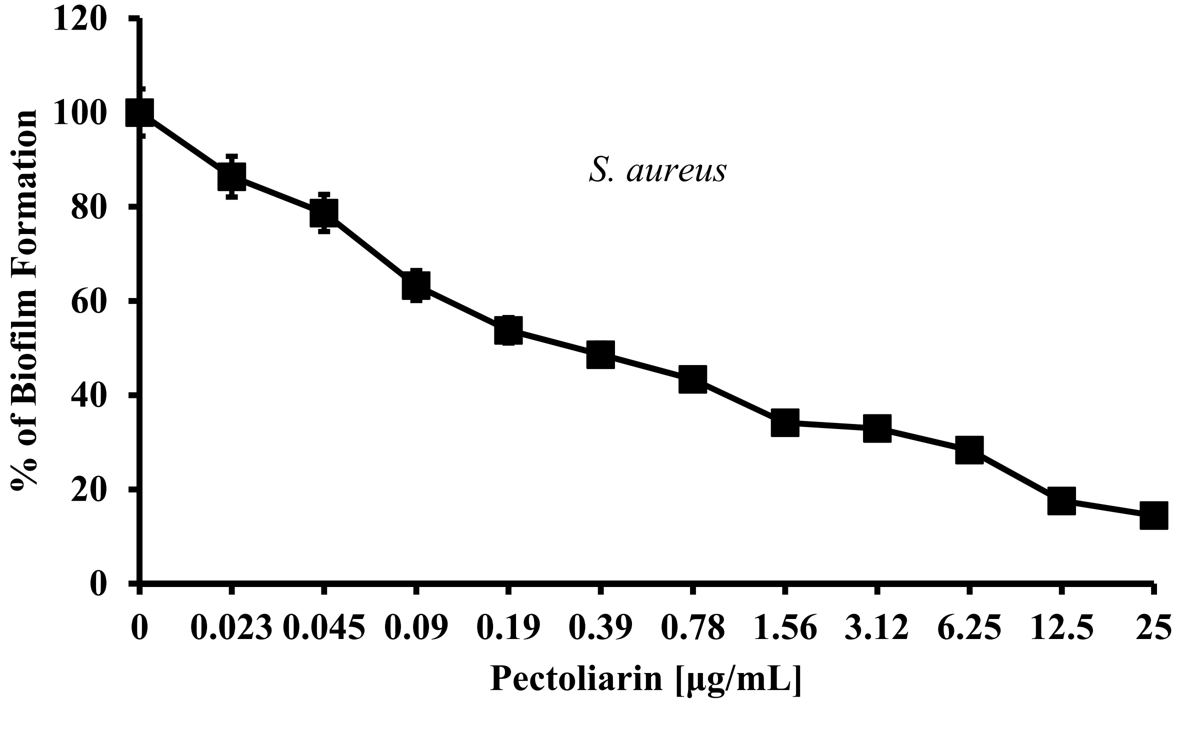

S. aureus
% of Biofilm Formation
120
100
80
60
40
20
0
0 0.023 0.045 0.09 0.19 0.39 0.78 1.56 3.12 6.25 12.5 25
Pectoliarin [µg/mL]

(e)

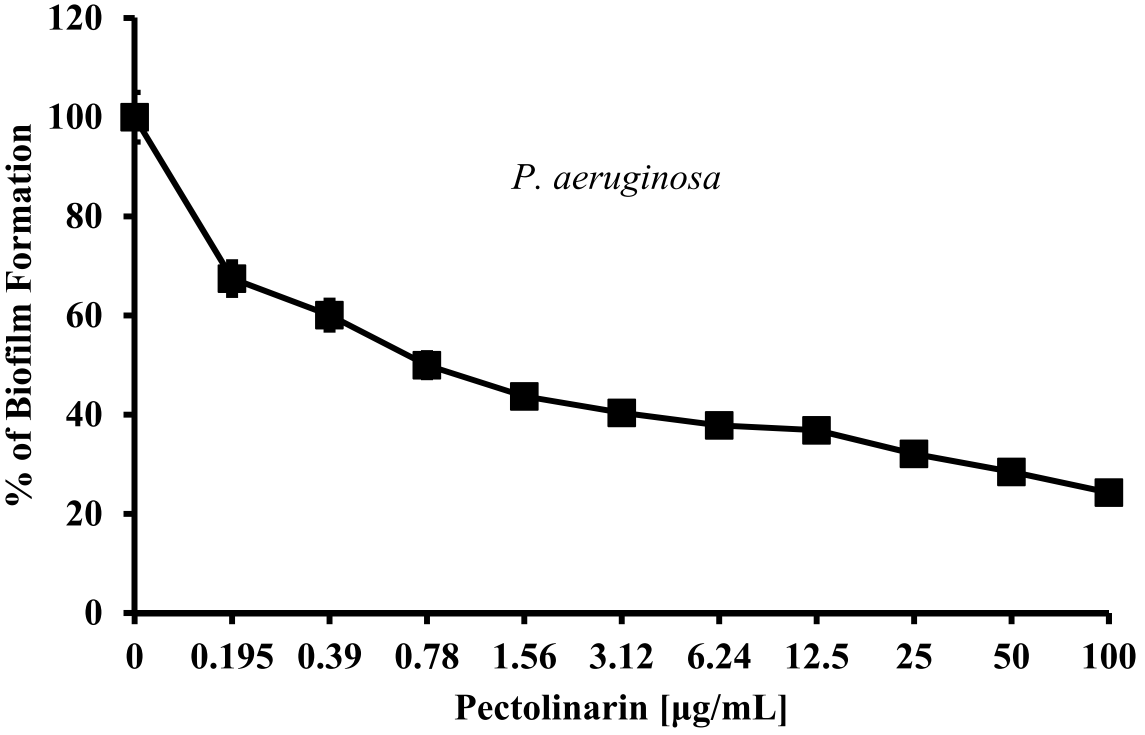
120
100
80
60
40
20
0
% of Biofilm Formation
P. aeruginosa
0
0.195
0.39
0.78
1.56
3.12
6.24
12.5
25
50
100
Pectolinarin [µg/mL]

(f)

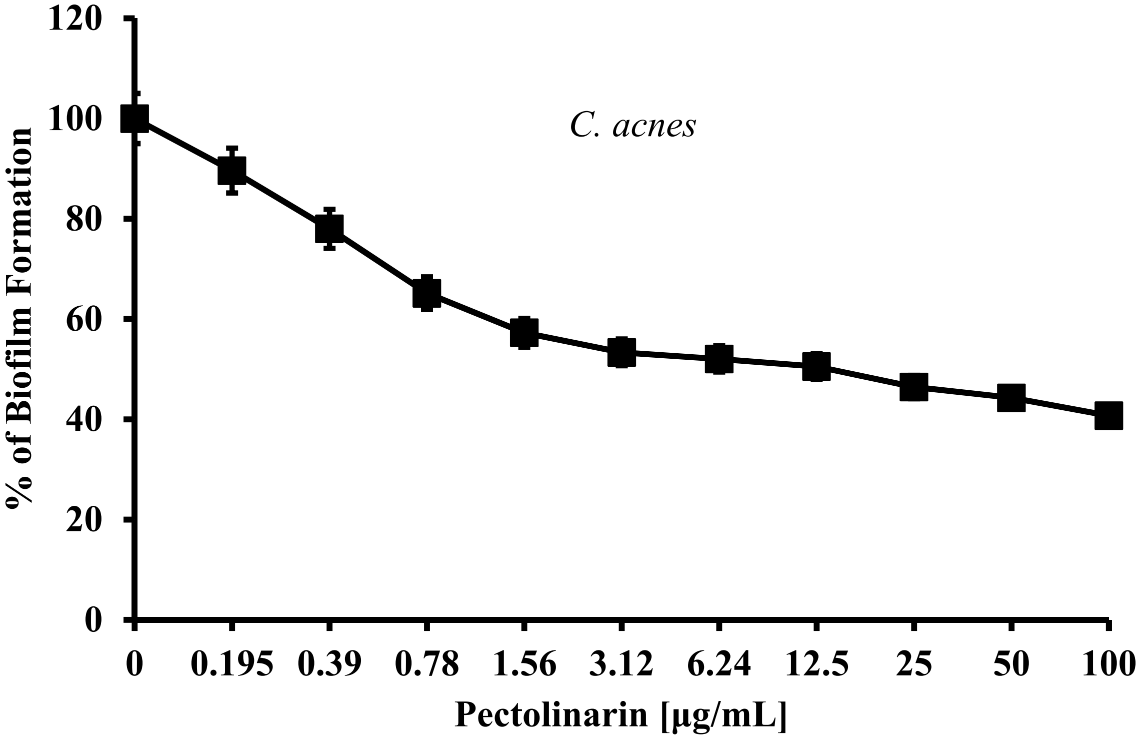
120
100
80
60
40
20
0
% of Biofilm Formation
C. acnes
0
0.195
0.39
0.78
1.56
3.12
6.24
12.5
25
50
100
Pectolinarin [µg/mL]

(g)

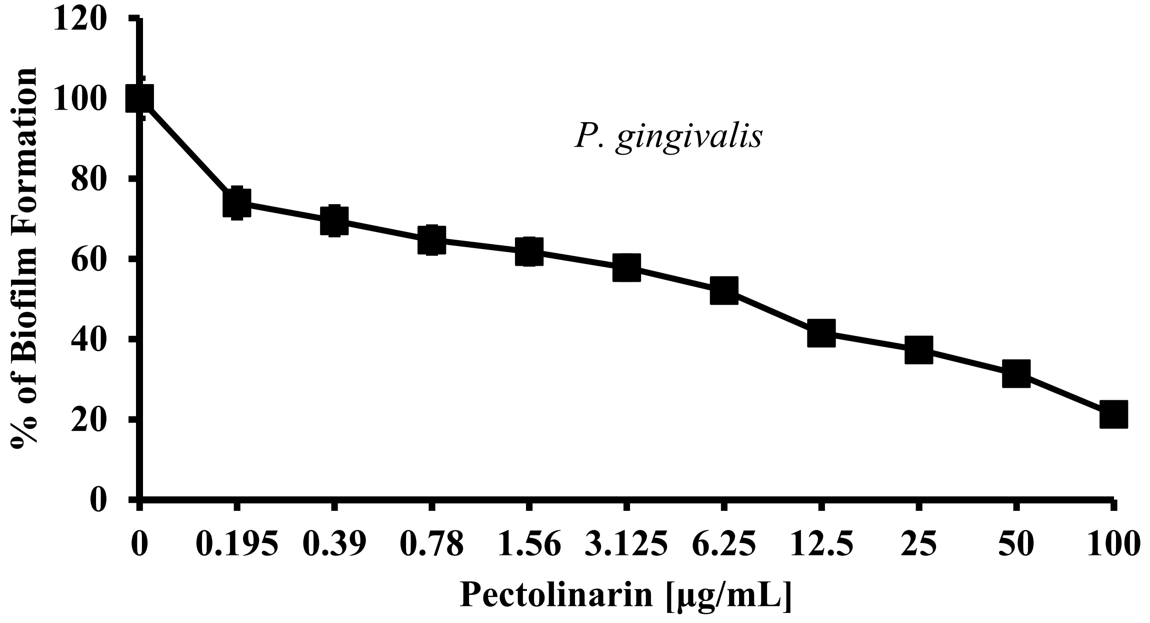


**Supplementary Figure S1.** Pectolinarin inhibited biofilm formation of (a) *E. coli*, (b) *S. mutans*, (c) *S. sobrinus*, (d) *S. aureus*, (e) *P. aeruginosa*, (f) *C. acnes* and (g) *P. gingivalis*. Biofilm of *E. coli*, *S. mutans*, *S. sobrinus*, *S. aureus*, *P. aeruginosa*, *C. acnes* and *P. gingivalis* was formed in medium supplemented with pectolinarin at the indicated concentrations at 37℃ for 24h.
